# Insulin resistance in the liver: Deficiency or excess of insulin?

**DOI:** 10.4161/15384101.2014.947750

**Published:** 2014-10-30

**Authors:** Roberto B Bazotte, Lorena G Silva, Fabiana PM Schiavon

**Affiliations:** Department of Pharmacology and Therapeutics; State University of Maringá; Maringá, Paraná, PR Brazil

**Keywords:** hyperinsulinism, insulin resistance, liver glucose production, liver lipogenesis, nonparenchymal liver cells, obesity, periportal hepatocytes, perivenous hepatocytes, type 2 diabetes

## Abstract

In insulin-resistant states (obesity, pre-diabetes, and type 2 diabetes), hepatic production of glucose and lipid synthesis are heightened in concert, implying that insulin deficiency and insulin excess coexists in this setting. The fact that insulin may be inadequate or excessive at any one point in differing organs and tissues has many biologic ramifications. In this context the concept of metabolic compartmentalization in the liver is offered herein as one perspective of this paradox. In particular, we focus on the hypothesis that insulin resistance accentuates differences in periportal and perivenous hepatocytes, namely periportal glucose production and perivenous lipid synthesis. Subsequently, excessive production of glucose and accumulation of lipids could be expected in the livers of patients with obesity and insulin resistance. Overall, in this review, we provide our integrative perspective regarding how excessive production of glucose in periportal hepatocytes and accumulation of lipids in perivenous hepatocytes interact in insulin resistant states.

Development of insulin resistance with obesity, pre-diabetes, and type 2 diabetes is a physiopathologic process where cells fail to respond normally to insulin.^1-4^ Thus, suppression of glucose production in the liver is decreased and activation of GLUT-4-mediated glucose uptake does not take place, particularly in skeletal muscles and adipocytes.^5^ This overall failure typically is not due to low insulin levels.^6^ Instead, insulin-stimulated signal transduction pathways for peripheral glucose uptake and for hepatic glucose production are reduced, including insulin receptors and downstream mediators.^7-9^

Hyperglycemia is then driven by excessive hepatic glucose production^10,11^ and reduced uptake of glucose by peripheral tissues.^12^ To counteract resultant glycemic elevations, β cells of the pancreas boost insulin production, further contributing to hyperinsulinemia.^12,13^ Hence, insulin resistance often is accompanied by increased circulating levels of insulin.^14-16^ If this compensatory rise in insulin production is not maintained by the pancreas, causing insulin levels to drop, then type 2 diabetes ensues.^17-19^

## Questions Raised by Insulin Resistance

It is well recognized that lipolysis and weight loss are accelerated in the absence of insulin, underscoring the fact that insulin stimulates lipogenesis,^20^ and raising an important question: why are about 90% of patients with type 2 diabetes overweight or obese?^21^ Or otherwise stated, is there a correlation between insulin deficiency and the tendency gain weight? Furthermore, given the scope of lipid deposition in liver (hepatic steatosis), skeletal muscle (intramyocytic lipid accumulation), cardiac muscle, and adipose tissues that is seen with insulin resistance,^22^ one must also ask: is obesity the cause or the result of insulin resistance?

The term insulin resistance oversimplifies a highly complex physiopathologic process for which a single overarching mechanism is not easily conceived.^1-6^ In addition, the paradigm that insulin resistance is pathologic at all times is simply inaccurate. For example, during late pregnancy, insulin resistance and increased glucose tolerance may be seen together as a seeming paradox.^23^ Nonetheless, some degree of insulin resistance during late pregnancy is necessary for glucose maintenance,^13^ owing to substantial fetal demands for glucose.

These questions and considerations have spawned a number of questions where answers may be found or conclusions drawn.

## Which Came First: The Chicken or the Egg?

Under physiologic conditions, the insulin sensitivity of various bodily tissues differs. Case in point, human skeletal muscle is more sensitive than subcutaneous fatty in terms of the effects of circulating insulin.^24^

Similarly, insulin resistance in insulin sensitive cells is not uniform but is tissue specific.^16^ For example, in high fat diet fed rats, insulin resistance was found to initiate in the liver, prior to developing in skeletal muscle.^26^ In addition, impairment in all steps of insulin signaling was detected in skeletal muscle, liver, hypothalamus, but not in adipose tissue of fat-rich diet treated mice.^12^ Hence, even in insulin-resistant states in which glucose transport is impaired, sensitivity to insulin's antilipolytic effect is relatively preserved, resulting in maintenance or expansion of adipose stores.

Thus, insulin resistance is not a synchronous, all-or-nothing process but rather builds in select organs or tissues amidst normal insulin response.

In parts of the body responding normally to insulin, the hyperinsulinism of insulin resistance likely is construed as a state of insulin excess. Hence, the catabolic activities of insulin resistance (increased hepatic glucose production, decreased glucose uptake, fasting hyperglycemia) may collide with complementary conditions that favor lipogenesis and obesity. So a vicious cycle can be set up with insulin resistance promoting weight gain, which promotes more insulin resistance. Subsequently, the multiple comorbidities of obesity, prediabetes, and type 2 diabetes that are attributed to insulin deficiency may actually stem in part from insulin excess. Likewise, the predisposition in patients with insulin resistance for aging,^1,18,27^ cancer,^27^ liver steatosis,^28^ dyslipidemia,^5^ atherosclerosis,^29^ cardiovascular disease,^30^ and why intensive insulin therapy may initially worsen retinophaty,^31^ also be at least partially explained.

## Coexistent Insulin Deficiency and Insulin Excess in Organs and Tissues of the Body

Development of hyperglycemia in overweight patients,^21^ is also aligned with the concept that throughout the body, both insulin deficiency and insulin excess are operant in insulin resistance. Indeed, these 2 extremes of response are even displayed by the same organ, i.e., increased hepatic production of glucose,^12,25^ and parenchymal deposition of lipid,^32-36^ associated with insulin resistance,^37,38^ suggesting that this principle could be applied to organs and tissues separately, as well as involving the body as a whole.

Interestingly, with insulin resistance induced by a high-fat diet, a temporal sequence has been noted for each substrate during activation of hepatic gluconeogenesis, with progressive intensification for L-lactate, glycerol, and alanine on days 7, 14, and 56, respectively after dietary implementation.^25^ If intensified liver gluconeogenesis then serves as a marker of insulin resistance, it appears that this process is quite specific, marked by intra-organ metabolic pathways that are unique for each substrate.

At this juncture, a new question surfaces: as with organs and tissues and the body as a whole, is the coexistence of insulin deficiency and insulin excess in insulin-resistant states applicable to isolated cells?

## Metabolic Compartmentalization of Glucose Production and Lipid Synthesis in Liver

To our knowledge, a number of metabolic pathways (i.e., gluconeogenesis, lipogenesis, glycolysis, glycogenolysis, ureagenesis, ketogenesis, synthesis and catabolism of amino acids, etc.) are feasible in individual hepatocytes. Hence, gluconeogenesis (a process inhibited by insulin) and lipogenesis (which is stimulated by insulin) are achievable in the same liver cell. However, in the acini area, the hepatocytes are exposed to a spatial biochemical gradient that influences metabolism and gene expression, so cell specialization does exist to some degree, depending on locale.^37-52^

**Figure 1.**
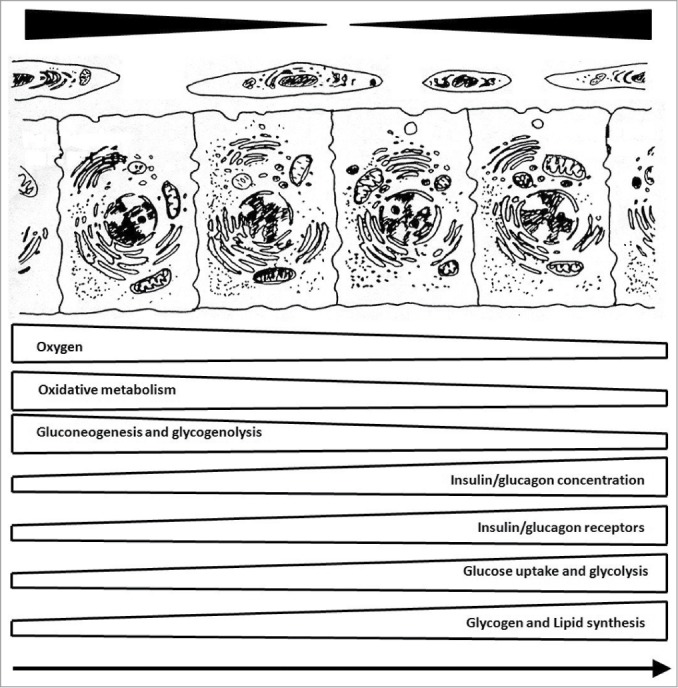
Functional compartmentalization of liver: periportal hepatocytes on left and perivenous hepatocytes on right, with arrow in direction of blood flow.

Parenchymal acini of the liver are divisible into 2 circulatory zones, based on proximity to afferent vessels.^36^ Periportal hepatocytes are supplied by blood rich in oxygen and nutrients, whereas the blood reaching perivenous hepatocytes (at the periphery of acini) is oxygen-poor and nutrient-depleted.^53,54^ This distinctive microvascular arrangement encourages metabolic heterogeneity. Periportal hepatocytes, harboring an abundance of mitochondria and sympathetic nerves are ideally suited for oxidative metabolism or glucose production, and perivenous hepatocytes are optimally configured for anaerobic metabolism and lipid synthesis (**Fig. 1**).

Hepatocytes are also subject to differential regulatory control, due to gradients in oxygen, substrate, and hormone levels. Notably, O_2_ partial pressure of approximately 65 mmHg in periportal areas drops to 35 mmHg in perivenous zones.^52^ The O_2_ partial pressure regulates the expression of genes encoding glucose-metabolizing enzymes, (for example: pyruvate carboxykinase, glucokinase and pyruvate kinase), through O_2_-responsive transcription factors, such as hypoxia-inducible factor (HIF).^53,54^ Moreover, liver metabolism is controlled among others by nuclear receptors,^36^ mammalian target of rapamycin (mTOR) pathway,^37^ and sirtuin family of proteins.^38^

The model of metabolic zonation assumes a functional specialization by each hepatic zone (**Fig. 1**). In periportal areas, gluconeogenesis, glycogenolysis, β-oxidation, amino acid metabolism, ureagenesis, and uric acid production predominate,^55-61^ reserving lipogenesis, glycolysis, glutaminogenesis, and biotransformation for perivenous areas.^62-72^ Accordingly, periportal and perivenous acinar zones differ in content of many key enzymes and subcellular constituents (**Table 1A** and **Table 1B**), all of which serve to optimize liver function for a central role in metabolic homeostasis.^36^

**Table 1A.** Zonation of cells, receptors, metabolism and biotransformation in liver. Key: ^+++^ predominant localization in periportal or perivenous zone.

	Periportal zone	Perivenous zone
Oxygen gradient^53,54^	+++	
Cell size^41^	15-20 μm	30-40 μm
Kupffer cells^39,40^	Phagocytosis	Cytotoxicity
Endothelial cells - Fenestrae^39,40^	Larger	Smaller
Stellate cells and Pit cells^39,40^	+++	
Sympathetic nerves^50^	+++	
Glucagon receptors^75^	+++	
Insulin receptors^52^		+++
Insulin/glucagon levels^76^		+++
Mitochondria and aerobic metabolism^36^	+++	
Glucose uptake and glycolysis^50^		+++
Glucose release: gluconeogenesis^36^	+++	
Glucose release: glycogenolysis^56^	+++	
ß-oxidation and ketogenesis^50^	+++	
Peroxisomal lipid oxidation^70^		+++
Triglycerides^59^ and VLDL synthesis^83^		+++
Cholesterol and bile synthesis^71^	+++	
Glycogen synthesis from glucose^59^		+++
Glycogen synthesis from pyruvate and lactate^50^	+++	
Uptake of the majority of amino acids^70^	+++	+++
Uptake of glutamate and aspartate^70^		
Uptake of α-ketoglutarate and malate^70^	+++	+++
Glutamine synthesis and release^60^		
Amino acid catabolism and urea synthesis^59^	+++	
Uric acid synthesis from adenosine^57^	+++	
Glutation peroxidase and ROS detoxification^50^	+++

**Table 1B.** Zonation of enzyme activity and protein synthesis in liver. Key: ^+++^ predominant localization in periportal or perivenous zone.

	**Periportal zone**	**Perivenous zone**
Phosphoenolpyruvate carboxykinase^50^	+++	
Pyruvate carboxykinase^41^	+++	
Fructose-1,6-biphosphatase^72^	+++	
Glucose-6-Phosphatase^58^	+++	
Pyruvate kinase type L^59^ and glukokinase^76^		+++
Acetyl-CoA carboxylase^83^		+++
Suppressor of cytokine signaling 2 (SOCS-2)^68^		+++
Glutaminase^60^	+++	
Glutamine synthetase^48^		+++
Succinate dehydrogenase^58^	+++	
Hydroximethylglutaryl-Coa-reductase^59^	+++	
Alanine^48^ and tyrosine aminotransferase^59^	+++	
Carbamoyl phosphate synthetase^59^	+++	
UDP-glucoronosyltransferase^50^		+++
Cytocrome P-450^45^		+++
Serine dehydratase^61^	+++	
Fibrinogen and laminin synthesis^50^	+++	
α2-macroglobulin and conexin 26 synthesis^50^	+++	
Collagen IV and V synthesis^41^	+++	
Collagen I, III and VI synthesis^41^		+++
α1-antitrypsin and fibronectin synthesis^50^		+++
α-fetoprotein and angiotensinogen synthesis^50^		+++
Lectin binding^62^		+++
Heme synthesis^46^		+++
Gene expression of albumin^48^		+++
Xenobiotic metabolism^67^		+++

It must be emphasized that functional specialization of this nature is also quite flexible. For example, the periportal-to-perivenous ratios for mitochondrial palmitate oxidation in fed, starved, re-fed, and cold-exposed animals were 1.5, 2.0, 1.0, and 0.4, respectively.^73^

Given that insulin released by β cells of the pancreas reaches periportal zones first, greater inhibition of glucose production in periportal area and lesser activation of lipogenesis perivenous area are anticipated.^74^ However, insulin receptors are predominantly found in the perivenous zone, where their expression is enhanced by high glucose concentration and decreased venous partial pressure.^52^ By contrast, glucagon receptors predominate in the periportal zone,^75^ where glucose release from glycogenolysis and gluconeogenesis preferentially takes place. In addition, due to biotransformation, glucagon and insulin concentrations decline during a single passage of blood through the liver by approximately 50% and 15%, respectively, resulting in proportionately higher perivenous concentrations of insulin.^50,75^

Therefore, under physiologic conditions, glucagon and insulin (from pancreatic α and β cells, respectively) first reach periportal zones, where glucagon receptors predominate for glycogenolysis and gluconeogenesis.^76^ Because after meal the biotransformation of glucagon is comparatively more rapid,^75^ the insulin/glucagon ratio increases as blood circulates to the periphery of liver acini. The higher insulin/glucagon ratio of perivenous zones, in conjunction with their preponderance of insulin receptors, is then favorable for lipid synthesis.

Here again, a question can be raised: is the paradox of insulin resistance, namely the hyperproduction of glucose in liver alongside steatosis, adequately explained by the concept of metabolic compartmentalization?

## Hepatic Glucose Hyperproduction and Steatosis in Insulin Resistance

Liver metabolism comprises an immense spectrum of interrelated anabolic and catabolic functions which are performed simultaneously without futile cycles. Therefore, functional compartmentalization of the liver, as shown in **Fig. 1**, implies coexistence of an anabolic liver in a fed state (with perivenous insulin effects predominating) and a catabolic liver in a fasted state (with predominance of periportal glucagon effects).

Interestingly, decreasing periportal-perivenous gradients of oxygen tension and increasing periportal-perivenous gradients of insulin: glucagon ratio appear to be major factors in the zonation,^50^ but how is functional compartmentalization of the liver affected by insulin resistance?

**Figure 2.**
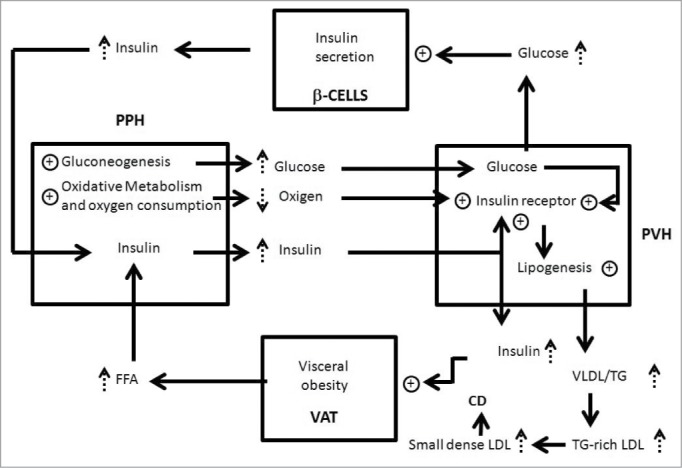
Relationship between visceral obesity, insulin resistance, functional compartmentalization and liver steatosis. Visceral adipocytes (VAT), periportal hepatocytes (PPH), perivenous hepatocytes (PVH) and β cells of pancreas (β-CELLS) are represented by squares. Key:↑augmentation; ⊕ stimulation; cardiovascular disease (CV); low-density lipoproteins (LDL); very low density lipoprotein (VLDL); triglyceride (TG).

A schematic of our theories is shown in **Fig. 2**, starting with an increased basal rate of lipolysis as a consequence of augmented visceral adiposity.^22,77^

Because insulin stimulates expansion of visceral fat, this pathologic process functions as a self-sustaining closed-loop system that will only be interrupted by weight loss (diet and/or exercise) and /or use of drugs to increase insulin sensitivity.

The excess of free fatty acids (FFA) from splanchnic lipolysis is taken up by periportal hepatocytes (**Fig. 2**) and oxidized as a source of energy, all of which biochemically increases oxygen consumption.^78^

In spite of the fact that FFA cannot be used as substrates for gluconeogenesis, their oxidation furnishes energy to increase glucose production via gluconeogenesis. In addition, the excess of FFA delivery to the liver, results in accumulation of intracellular diacyglycerols which, in turn, leads to activation of protein kinase C (PKC). PKC induce insulin resistance by inhibiting insulin-stimulated phosphorylation of IRS proteins. Furthermore the excess of FFA results in activation of inflammatory toll-like receptors (TLR) signaling leading to increased *de novo* ceramide synthesis. Thus, accumulation of ceramides and ceramide-mediated inhibition induce insulin resistance in the liver by inhibition of Akt phosphorylation.^79^ Yet, FFA can cause activation of Akt phosphatase protein phosphatase 2A (PP2A). PP2A induce insulin resistance in the liver by dephosphorilation and inactivation of Akt that in turn acts to phosphorylate and inactivate the transcription factor Forkhead Box 01 (FOX-01), which induces transcription of the key enzimes of gluconeogenesis.^80^

As consequence of these metabolic changes,^73^ perivenous hepatocytes exposed to increased glucose concentration and reduced levels of oxygen,^53^ intensifying their expression of insulin receptors.^52^

The enhanced FFA also increase the synthesis of triglyceride (TG) and very-low-density lipoprotein (VLDL) production in the perivenous zone,^81-83^ thereby promoting hypertriglyceridemia. Furthermore, the TG in VLDL is exchanged for cholesteryl esters from low-density lipoproteins (LDL) and high-density lipoproteins (HDL) by the cholesteryl ester transport protein, producing TG-rich LDL and TG-rich HDL. The TG in the TG-rich LDL and TG-rich HDL is then hydrolyzed by hepatic lipase, producing small dense LDL and small dense HDL. The formation of these particles are linked to a higher risk of cardiovascular disease.^5^ In fact, patients die more often from cardiovascular disease than from direct consequences of liver steatosis.^84^

## Concluding Remarks

All aspects of the human body must be considered to better understand the mechanisms of insulin resistance and to appreciate how insulin deficiency and insulin excess may coexist in this pathologic condition. The fact that insulin may be inadequate or excessive at any one point in differing organs and tissues has many biologic ramifications. Ultimately, attention must be paid to specific defects of insulin signaling in isolated cells and to subcellular compartments affected by the increased circulating insulin that accompanies insulin resistance. However, despite the enthusiasm for molecular aspects, we must never lose sight of the isolated organ and *in vivo* situation.

One key message is that insulin resistance accentuates differences in periportal and perivenous hepatocytes, namely periportal glucose production and perivenous lipogenesis. Subsequently, excessive production of glucose and accumulation of lipids are to be expected in the livers of patients with obesity and insulin resistance.

However, there is still a great deal to be learned about the mechanisms linking insulin resistance and metabolic compartmentalization in the liver. Therefore, future studies on the regulation of zonal gene expression in parenchymal and nonparenchymal liver cells will provide advances in our understanding of the impact of insulin resistance on metabolic compartmentalization in the liver.

## References

[1] Blagosklonny MV. TOR-centric view on insulin resistance and diabetic complications: perspective for endocrinologists and gerontologists. Cell Death Dis 2013; 4:e964; PMID:24336084; http://dx.doi.org/10.1038/cddis.2013.506 24336084 PMC3877573

[2] Hirabara SM, Gorjão SM, Vinolo MA, Rodrigues AC, Nachbar RT, Curi R. Molecular targets related to inflammation and insulin resistance and potential interventions. J Biomed Biotechnol 2012; 2012:379024; PMID:23049242; http://dx.doi.org/10.1155/2012/379024 23049242 PMC3463198

[3] Park SY, Cho YR, Kim HJ, Higashimori T, Danton C, Lee MK, Dey A, Rothermel B, Kim YB, Kalinowski A, et al. Unraveling the temporal pattern of diet-induced insulin resistance in individual organs and cardiac dysfunction in C57BL/6 mice. Diabetes 2005; 54:3530-40; PMID:16306372; http://dx.doi.org/10.2337/diabetes.54.12.3530 16306372

[4] Li H, Zhou B, Xu L, Liu J, Zang W, Wu S, Sun H. The reciprocal interaction between autophagic dysfunction and ER stress in adipose insulin resistance. Cell Cycle 2014; 13(4):565-79; PMID:24309597; http://dx.doi.org/10.4161/cc.27406 24309597

[5] Jung UJ, Choi MS. Obesity and its metabolic complications: the role of adipokines and the relationship between obesity, inflammation, insulin resistance, dyslipidemia and nonalcoholic fatty liver disease. Int J Mol Sci 2014; 15:6184-223; PMID:24733068; http://dx.doi.org/10.3390/ijms15046184 24733068 PMC4013623

[6] Knights AJ, Funnell AP, Pearson RC, Crossley M, Bell-Anderson KS. Adipokines and insulin action: a sensitive issue. Adipocyte 2014; 3:88-96; PMID:24719781; http://dx.doi.org/10.4161/adip.27552 24719781 PMC3979885

[7] Cai D. NFkappaB-mediated metabolic inflammation in peripheral tissues versus central nervous system. Cell Cycle 2009; 8:2542-48; PMID:19633416; http://dx.doi.org/10.4161/cc.8.16.9386 19633416

[8] Perry RJ, Shulman GI Treating fatty liver and insulin resistance. Aging 2013; 5(11):791-2; PMID:2430466224284975 24304662 10.18632/aging.100617 PMC3868721

[9] Hotamisligil GS Inflammation and metabolic disorders. Nature 2006; 444:860-67; PMID:17167474; http://dx.doi.org/24284975 10.1038/nature05485 17167474

[10] Barrena HC, Schiavon FP, Cararra MA, Marques A de C, Schamber CR, Curi R, Bazotte RB. Effect of linseed oil and macadamia oil on metabolic changes induced by high-fat diet in mice. Cell Biochem Funct 2014; 32(4):333-40; PMID:24284975; http://dx.doi.org/10.1002/cbf.3018 24284975

[11] Weyer C, Bogardus C, Mott DM, Pratley RE The natural history of insulin secretory dysfunction and insulin resistance in the pathogenesis of type 2 diabetes mellitus. J Clin Invest 1999; 104:787-94 PMID:10491414; http://dx.doi.org/17761768 10.1172/JCI7231 10491414 PMC408438

[12] Araújo EP, De Souza CT, Ueno M, Cintra DE, Bertolo MB, Carvalheira JB, Saad MJ, Velloso LA. Infliximab restores glucose homeostasis in an animal model of diet-induced obesity and diabetes. Endocrinology 2007; 148:5991-97; PMID:17761768; http://dx.doi.org/10.1210/en.2007-0132 17761768

[13] Jacovetti C, Regazzi R. Compensatory β-cell mass expansion: a big role for a tiny actor. Cell Cycle 2013; 12(2):197-8; PMID:23287464; http://dx.doi.org/10.4161/cc.23378 23287464 PMC3575443

[14] Wu M, Wang X, Duan Q, Lu T Arachidonic acid can significantly prevent early insulin resistance induced by a high-fat diet. Ann Nutr Metab 2007; 51:270-76; PMID:17622786; http://dx.doi.org/21483037 10.1159/000105448 17622786

[15] Vetterli L, Maechler P. Resveratrol-activated SIRT1 in liver and pancreatic β-cells: a Janus head looking to the same direction of metabolic homeostasis. Aging 2011; 3(4):444-9; PMID:21483037 21483037 10.18632/aging.100304 PMC3117460

[16] Lim S, Son KR, Song IC, Park HS, Jin CJ, Jang HC, Park KS, Kim YB, Lee HK. Fat in liver/muscle correlates more strongly with insulin sensitivity in rats than abdominal fat. Obesity 2009; 17:188-95; PMID:18948962; http://dx.doi.org/10.1038/oby.2008.486 18948962

[17] DeFronzo RA, Banerji MA, Bray GA, Buchanan TA, Clement S, Henry RR, Kitabchi AE, Mudaliar S, Musi N, Ratner R, et al. Determinants of glucose tolerance in impaired glucose tolerance at baseline in the actos now for prevention of diabetes (ACT NOW) study. Diabetologia 2010; 53:435-45; PMID:20012012; http://dx.doi.org/10.1007/s00125-009-1614-2 20012012

[18] Blagosklonny MV Once again on rapamycin-induced insulin resistance and longevity: despite of or owing to. Aging 2012; 4(5):350-8; PMID:2268366122542394 22683661 10.18632/aging.100461 PMC3384435

[19] Kahn SE The relative contributions of insulin resistance and beta-cell dysfunction to the pathophysiology of type 2 diabetes. Diabetologia 2003; 46:3-19; PMID:12637977; http://dx.doi.org/22542394 10.1007/s00125-003-1190-9 12637977

[20] Thorens B The required beta cell research for improving treatment of type 2 diabetes. J Intern Med 2013; 274:203-14; PMID:23751050; http://dx.doi.org/22542394 10.1111/joim.12096 23751050

[21] Kumanyika S, Jeffery RW, Morabia A, Ritenbaugh C, Antipatis VJ Obesity prevention: the case for action. Int J Obe Relat Metab Disord 2002; 26:425-36; PMID:11896500; http://dx.doi.org/22542394 10.1038/sj.ijo.0801938 11896500

[22] Ravussin E, Smith SR Increased fat intake, impaired fat oxidation, and failure of fat cell proliferation result in ectopic fat storage, insulin resistance, and type 2 diabetes mellitus. Ann N Y Acad Sci 2002; 967:363-78; PMID:12079864; http://dx.doi.org/22542394 10.1111/j.1749-6632.2002.tb04292.x 12079864

[23] Carrara MA, Batista MR, Saruhashi TR, Felisberto AM Jr, Guilhermetti M, Bazotte RB. Coexistence of insulin resistance and increased glucose tolerance in pregnant rats: a physiological mechanism for glucose maintenance. Life Sci 2102; 90:831-7; PMID:22542394; http://dx.doi.org/10.1016/j.lfs.2012.03.037 22542394

[24] Stumvoll M, Jacob S, Wahl HG, Hauer B, Löblein K, Grauer P, Becker R, Nielsen M, Renn W, Häring H. Suppression of systemic, intramuscular, and subcutaneous adipose tissue lipolysis by insulin in humans. J Cli Endocrino Metab 2000; 85:3740-5; PMID:11061533; http://dx.doi.org/10.1210/jcem.85.10.6898 11061533

[25] Obici S, Tavoni TM, Barrena HC, Curi R, Bazotte RB. Time sequence of the intensification of the liver glucose production induced by high-fat diet in mice. Cell Biochem Func 2012; 30:335-59; PMID:22315157; http://dx.doi.org/10.1002/cbf.2809 22315157

[26] Kraegen EW, Clark PW, Jenkins AB, Daley EA, Chisholm DJ, Storlien LH Development of muscle insulin resistance after liver insulin resistance in high-fat-fed rats. Diabetes 1991; 40:1397-403;PMID:1936601; http://dx.doi.org/24189526 10.2337/diab.40.11.1397 1936601

[27] Anisimov VN. Metformin: do we finally have an anti-aging drug? Cell Cycle 2013; 12:3483-9; PMID:24189526; http://dx.doi.org/10.4161/cc.26928 24189526 PMC3906334

[28] Bedogni G, Kahn HS, Bellentani S, Tiribelli CA. Simple index of lipid overaccumulation is a good marker of liver steatosis. BMC Gastroenterol 2010; 10:98; PMID:20738844; http://dx.doi.org/10.1186/1471-230X-10-98 20738844 PMC2940930

[29] DeFronzo RA. Insulin resistance, lipotoxicity, type 2 diabetes and atherosclerosis: the missing links. The Claude Bernard Lecture 2009. Diabetologia 2010; 53:1270-87; PMID:20361178; http://dx.doi.org/10.1007/s00125-010-1684-1 20361178 PMC2877338

[30] Dorn GW 2nd Mechanisms of non-apoptotic programmed cell death in diabetes and heart failure. Cell Cycle 2010; 9:3442-8; PMID:20814234; http://dx.doi.org/24810050 10.4161/cc.9.17.12944 20814234 PMC3230473

[31] Leontieva OV, Demidenko ZN, Blagosklonny MV. Rapamycin reverses insulin resistance (IR) in high-glucose medium without causing IR in normoglycemic medium. Cell Death Dis 2014; 5:e1214; PMID:24810050; http://dx.doi.org/10.1038/cddis.2014.178 24810050 PMC4047870

[32] Fruci B, Giuliano S, Mazza A, Malaguarnera R, Belfiore A. Nonalcoholic fatty liver: a possible new target for type 2 diabetes prevention and treatment. Int J Mol Sci 2013; 14:22933-66; PMID:24264040; http://dx.doi.org/10.3390/ijms141122933 24264040 PMC3856099

[33] Hooper AJ, Adams LA, Burnett JR Genetic determinants of hepatic steatosis in man. J Lipid Res 2011; 52:593-17; http://dx.doi.org/10.1194/jlr.R008896 21245030 PMC3053205

[34] Masuoka HC, Chalasani N. Nonalcoholic fatty liver disease: an emerging threat to obese and diabetic individuals. Ann N Y Acad Sci 2013; 1281:106-22; PMID:23363012; http://dx.doi.org/10.1111/nyas.12016 23363012 PMC3646408

[35] Sanal MG. The blind men ‘see’ the elephant-the many faces of fatty liver disease. World J Gastroentero 2008; 14:831-44; PMID:18240340; http://dx.doi.org/10.3748/wjg.14.831 18240340 PMC2687050

[36] Godoi P, Hewitt NJ, Albrecht U, Andersen ME, Ansari N, Bhattacharya S, Bode JG, Bolleyn J, Borner C, Böttger J, et al. Recent advances in 2D and 3D in vitro systems using primary hepatocytes, alternative hepatocyte sources and non-parenchymal liver cells and their use in investigating mechanisms of hepatotoxicity, cell signaling and ADME. Arch Toxicol 2013; 87:1315-530; PMID:23974980; http://dx.doi.org/10.1007/s00204-013-1078-5 23974980 PMC3753504

[37] Blagosklonny MV Rapamycin-induced glucose intolerance: hunger or starvation diabetes. Cell Cycle 2011; 10(24):4217-24; PMID:22157190; http://dx.doi.org/22761194 10.4161/cc.10.24.18595 22157190

[38] Halperin-Sheinfeld M, Gertler A, Okun E, Sredni B, Cohen HY. The tellurium compound, AS101, increases SIRT1 level and activity and prevents type 2 diabetes. Aging 2012; 4(6):436-47; PMID:22761194 22761194 10.18632/aging.100468 PMC3409680

[39] Bouwens L, De Bleser P, Vanderkerken K, Geerts B, Wisse E. Liver cell heterogeneity: functions of non-parenchymal cells. Enzyme 1992; 46:155-68; PMID:1289080 1289080 10.1159/000468782

[40] Burt AD, Path MRC, Le B, Balabaud C, Bioulac-Sage P Morphologic investigation of sinusoidal cells. Semin Liv Dis 1993; 13:21-38; PMID:8446906; http://dx.doi.org/22837346 10.1055/s-2007-1007335 8446906

[41] Bhatia SN, Toner M, Foy BD, Rotem A, O'Neil KM, Tompkins RG, Yarmush ML Zonal liver cell heterogeneity: effects of oxygen on metabolic functions of hepatocytes. Cell Eng 1996; 1:25-35; available from http://lmrt.mit.edu/publications/1996/Bhatia1996_JCellEng.pdf

[42] Gioli-Pereira L, Nascimento EA, Santos EL, Bracht A, Juliano MA, Pesquero JB, Borges DR, Kouyoumdjian M Fate of bradykinin on the rat liver when administered by the venous or arterial route. J Gastroenterol Hepatol 2005; 20:463-73; PMID:15740493; http://dx.doi.org/22837346 10.1111/j.1440-1746.2005.03580.x 15740493

[43] Kobayashi T, Saito Y, Ohtake Y, Maruko A, Yamamoto Y, Yamamoto F, Kuwahara Y, Fukumoto M, Fukumoto M, Ohkubo Y. Effect of aging on norepinephrine-related proliferative response in primary cultured periportal and perivenous hepatocytes. Am J Physiol 2012; 303:G861-9; PMID:22837346; 10.1152/ajpgi.00081.2012 22837346

[44] Lopez CH, Constantin J, Gimenes D, Suzuki-Kemmelmeier F, Bracht A. Heterogenic response of the liver parenchyma to ethanol studied in the bivascularly perfused rat liver. Mol Cell Biochem 2004; 258:155-62; PMID:15030180; http://dx.doi.org/10.1023/B:MCBI.0000012850.90719.6e 15030180

[45] Püschel GP, Jungermann K. Integration of function in the hepatic acinus: intercellular communication in neural and humoral control of liver metabolism. Prog Liver Dis 1994; 12:19-46; PMID:7746874 7746874

[46] Braeuning A, Schwarz M. Zonation of heme synthesis enzymes in mouse liver and their regulation by β-catenin and Ha-ras. Biol Chem 2010; 391:1305-13; PMID:20707599; http://dx.doi.org/10.1515/BC.2010.115 20707599

[47] Chalhoub E, Xie L, Balasubramanian V, Kim J, Belovich J. A distributed model of carbohydrate transport and metabolism in the liver during rest and high-intensity exercise. Ann Biomed Eng 2007; 35:474-91; PMID:17151925; http://dx.doi.org/10.1007/s10439-006-9217-2 17151925

[48] Racine L, Scoazec JY, Moreau A, Chassagne P, Bernuau D, Feldmann G. Distribution of albumin, alpha 1-inhibitor 3 and their respective mRNAs in periportal and perivenous rat hepatocytes isolated by the digitonin-collagenase technique. Biochem J 1995; 305:263-8; PMID:7826339; available from http://www.biochemj.org/bj/305/0263/3050263.pdf 7826339 10.1042/bj3050263 PMC1136458

[49] Kmieć Z. Cooperation of liver cells in health and disease. Adv Anat Embryol Cell Biol 2001; 161:1-151; PMID:11729749 11729749 10.1007/978-3-642-56553-3

[50] Jungermann K, Kietzmann T Zonation of parenchymal and nonparenchymal metabolism in liver. Annu Rev Nutr 1996; 16:179-203; http://dx.doi.org/10.1146/annurev.nu.16.070196.001143 8839925

[51] Stümpel F, Kucera R, Bazotte RB, Püschell GP. Loss of regulation by sympathetic hepatic nerves of liver metabolism and haemodynamics in chronically streptozotocin-diabetic rats. Diabetologia 1996; 39:161-5; PMID:8635667; http://dx.doi.org/10.1007/BF00403958 8635667

[52] Krones A, Kietzmann T, Jungermann K. Perivenous localization of insulin receptor protein in rat liver, and regulation of its expression by glucose and oxygen in hepatocyte cultures. Biochem J 2000; 348:433-8; PMID:10816439; http://dx.doi.org/10.1042/0264-6021:3480433 10816439 PMC1221083

[53] Kietzmann T, Dimova EY, Flügel D, Scharf JG. Oxygen: modulator of physiological and pathophysiological processes in the liver. Z Gastroenterol 2006; 44:67-6; PMID:16397842; http://dx.doi.org/10.1055/s-2005-858987 16397842

[54] Allen JW, Bhatia SN. Formation of steady-state oxygen gradients in vitro: application to liver zonation. Biotechnol Bioeng 2003; 82:253-62; PMID:12599251; http://dx.doi.org/10.1002/bit.10569 12599251

[55] Comar JF, Suzuki-Kemmelmeier F, Constantin J, Bracht A. Hepatic zonation of carbon and nitrogen fluxes derived from glutamine and ammonia transformations. J Biomed Sci 2010; 17:1-11; PMID:20055990; http://dx.doi.org/10.1186/1423-0127-17-1 20055990 PMC2843605

[56] Gimenes D, Constantin J, Comar JF, Kelmer-Bracht AM, Broetto-Biazon AC, Bracht A. Liver parenchyma heterogeneity in the response to extracellular NAD+. Cell Biochem Funct 2006; 24:313-25; PMID:15920702; http://dx.doi.org/10.1002/cbf.1228 15920702

[57] Fernandes TR, Suzuki-Kemmelmeier F, Ishii-Iwamoto EL, Constantin J, Bracht A. Regional heterogeneities in the production of uric acid from adenosine in the bivascularly perfused rat liver. Mol Cell Biochem 1999; 195:207-17; PMID:10395085; http://dx.doi.org/10.1023/A:1006957227649 10395085

[58] Jungermann K Zonation of metabolism and gene expression in liver. Histochem Cell Biol 1995; 103:81-91; PMID:7634156; http://dx.doi.org/1906044 10.1007/BF01454004 7634156

[59] Jungermann K, Katz N Functional specialization of different hepatocyte populations. Physiol Rev 1989; 69:708-64; PMID:26648261906044 2664826 10.1152/physrev.1989.69.3.708

[60] Newsholme P, Lima MM, Procopio J, Pithon-Curi TC, Doi SQ, Bazotte RB, Curi R Glutamine and glutamate as vital metabolites. Braz J Med Biol Res 2003; 36:153-63; PMID:12563517; http://dx.doi.org/1906044 10.1590/S0100-879X2003000200002 12563517

[61] Ogawa H, Pitot HC, Fujioka M Diurnal variation of the serine dehydratase mRNA level in rat liver. Arch Biochem Biophys 1994; 8:285-91; PMID:8311466; http://dx.doi.org/1906044 10.1006/abbi.1994.1040 8311466

[62] Barberá-Guillem E, Rocha M, Alvarez A, Vidal-Vanaclocha F. Differences in the lectin-binding patterns of the periportal and perivenous endothelial domains in the liver sinusoids. Hepatology 1991; 14(1):131-9; PMID:1906044; http://dx.doi.org/10.1002/hep.1840140122 1906044

[63] Gebhardt R Metabolic zonation of the liver: regulation and implications for liver function. Pharmacol Ther 1992; 53:275-54; PMID:1409850; http://dx.doi.org/7942280 10.1016/0163-7258(92)90055-5 1409850

[64] Gebhardt R, Gaunitz F, Mecke D. Heterogeneous (positional) expression of hepatic glutamine synthetase: features, regulation and implications for hepatocarcinogenesis. Adv Enzyme Regul 1994; 34:27-56; PMID:7942280; http://dx.doi.org/10.1016/0065-2571(94)90007-8 7942280

[65] Kietzmann T, Jungermann K. Modulation by oxygen of zonal gene expression in liver studied in primary rat hepatocyte cultures. Cell Biol Toxicol 1997; 13:243-55; PMID:9298245; http://dx.doi.org/10.1023/A:1007427206391 9298245

[66] Lopez CH, Suzuki-Kemmelmeier F, Constantin J, Bracht A. Zonation of the action of ethanol on gluconeogenesis and ketogenesis studied in the bivascularly perfused rat liver. Chem Biol Interact 2009; 177:89-95; PMID:18992231; http://dx.doi.org/10.1016/j.cbi.2008.09.035 18992231

[67] Kanamura S, Kanai K, Watanabe J Fine structure and function of hepatocytes during development. J Electron Microsc Tech 1990; 14(2):92-105; PMID:2406390; http://dx.doi.org/19470084 10.1002/jemt.1060140204 2406390

[68] Zellmer D, Sickinger S, Schmidt-Heck W, Guthke R, Gebhardt R. Heterogeneous expression of suppressor of cytokine signalling 2 (SOCS-2) in liver tissue. J Anat 2009; 215(2):176-183; PMID:19470084; http://dx.doi.org/10.1111/j.1469-7580.2009.01085.x 19470084 PMC2740965

[69] Traub O, Look J, Dermietzel R, Brümmer F, Hülser D, Willecke K. Comparative characterization of the 21-k_D_ and 26-k_D_ gap junction proteins in murine liver and cultured hepatocytes. J Cell Biol 1989; 108:1039-51; PMID:2537831; http://dx.doi.org/10.1083/jcb.108.3.1039 2537831 PMC2115368

[70] Häussinger D, Lamers WH, Moorman AF Hepatocyte heterogeneity in the metabolism of amino acids and ammonia. Enzyme 1992; 46:72-93; PMID:12890831289085 1289083 10.1159/000468779

[71] Groothuis GM, Meijer DK. Hepatocyte heterogeneity in bile formation and hepatobiliary transport of drugs. Enzyme 1992; 46:94-138; PMID:1289085 1289085 10.1159/000468780

[72] Eilers F, Modaressi S, Jungermann K. Predominant periportal expression of the fructose-1.6-bisphosphatase gene in rat liver: dynamics during the daily feeding rhythm and starvation-refeeding cycle. Histochemistry 1995; 103:293-300; PMID:7648405; http://dx.doi.org/10.1007/BF01457414 7648405

[73] Guzmán M, Bijleveld C, Geelen MJ. Flexibility of zonation of fatty acid oxidation in rat liver. Biochem J 1995; 311:853-60; PMID:7487941 7487941 10.1042/bj3110853 PMC1136079

[74] Wei K, Piecewicz SM, McGinnis LM, Taniguchi CM, Wiegand SJ, Anderson K, Chan CW, Mulligan KX, Kuo D, Yuan J, et al. A liver Hif-2α-Irs2 pathway sensitizes hepatic insulin signaling and is modulated by Vegf inhibition. Nat Med 2013; 19(10):1331-7; PMID:24037094; http://dx.doi.org/10.1038/nm.3295 24037094 PMC3795838

[75] Krones A, Kietzmann T, Jungermann K. Periportal localization of glucagon receptor mRNA in rat liver and regulation of its expression by glucose and oxygen in hepatocyte cultures. FEBS Lett 1998; 421:136-40; PMID:9468294; http://dx.doi.org/10.1016/S0014-5793(97)01556-1 9468294

[76] Probst I, Schwartz P, Jungermann K. Induction in primary culture of “gluconeogenic” and “glycolytic” hepatocytes resembling periportal and perivenous cells. Eur J Biochem 1982; 126:271-8; PMID:6751822; http://dx.doi.org/10.1111/j.1432-1033.1982.tb06775.x 6751822

[77] Després JP, Lemieux I Abdominal obesity and metabolic syndrome. Nature 2006; 444:881-7; PMID:17167477; http://dx.doi.org/19531645 10.1038/nature05488 17167477

[78] Hue L, Taegtmeyer H. The Randle cycle revisited: a new head for an old hat. Am J Physiol Endocrinol Metab 2009; 297:E578-91; PMID:19531645; http://dx.doi.org/10.1152/ajpendo.00093.2009 19531645 PMC2739696

[79] Galbo T, Shulman GI Lipid-induced hepatic insulin resistance. Aging 2013; 5(8):582-3; PMID:2392989324150286 23929893 10.18632/aging.100585 PMC3796207

[80] Galbo T, Perry RJ, Nishimura E, Samuel VT, Quistorff B, Shulman GI. PP2A inhibition results in hepatic insulin resistance despite Akt2 activation. Aging 2013; 5(10):770-81; PMID:24150286; available from http://www.ncbi.nlm.nih.gov/pmc/articles/PMC3838779/24150286 10.18632/aging.100611 PMC3838779

[81] Nielsen S, Guo Z, Johnson CM, Hensrud DD, Jensen MD Splanchnic lipolysis in human obesity. J Clin Invest 2004; 113:1582-8; PMID:419492; http://dx.doi.org/24587651 10.1172/JCI21047 15173884 PMC419492

[82] Abdelmalek MF, Diehl AM Nonalcoholic fatty liver disease as a complication of insulin resistance. Med Clin North Am 2007; 91:1125-49; PMID:17964913; http://dx.doi.org/24587651 10.1016/j.mcna.2007.06.001 17964913

[83] Guzmán M, Castro J Zonation of fatty acid metabolism in rat liver. Biochem J 1989; 264:107-13 2574974 10.1042/bj2640107 PMC1133553

[84] Ballestri S, Lonardo A, Bonapace S, Byrne CD, Loria P, Targher G. Risk of cardiovascular, cardiac and arrhythmic complications in patients with non-alcoholic fatty liver disease. World J Gastroenterol 2014; 20:1724-45; PMID:24587651; http://dx.doi.org/10.3748/wjg.v20.i7.1724 24587651 PMC3930972

